# Instrumental Techniques for Characterization of Molybdenum Disulphide Nanostructures

**DOI:** 10.1155/2020/8896698

**Published:** 2020-12-16

**Authors:** Kabelo E. Ramohlola, Emmanuel I. Iwuoha, Mpitloane J. Hato, Kwena D. Modibane

**Affiliations:** ^1^Nanotechnology Research Lab, Department of Chemistry, School of Physical and Mineral Sciences, University of Limpopo (Turfloop), Sovenga 0727, Polokwane, South Africa; ^2^SensorLab, Chemistry Department, University of the Western Cape, Bellville, 7535 Cape Town, South Africa

## Abstract

The excellent chemical and physical properties of materials (nanomaterials) with dimensions of less than 100 nm (nanometers) resulted in researchers and industrialists to have great interest in their discovery and applications in various systems/applications. As their sizes are reduced to nanoscale, these nanomaterials tend to possess exceptional properties differing from those of their bulk counterparts; hence, they have found applications in electronics and medicines. In order to apply them in those applications, there is a need to synthesise these nanomaterials and study their structural, optical, and electrochemical properties. Among several nanomaterials, molybdenum disulphide (MoS_2_) has received a great interest in energy applications due to its exceptional properties such as stability, conductivity, and catalytic activities. Hence, the great challenge lies in finding the state-of-the-art characterization techniques to reveal the different properties of MoS_2_ nanostructures with great accuracy. In this regard, there is a need to study and employ several techniques to accurately study the surface chemistry and physics of the MoS_2_ nanostructures. Hence, this review will comprehensively discuss a detailed literature survey on analytical techniques that can be used to study the chemical, physical, and surface properties of MoS_2_ nanostructures, namely, ultraviolet-visible spectroscopy (UV-vis), photoluminescence spectroscopy (PL), Fourier transform infrared spectroscopy (FTIR), Raman spectroscopy, time-of-flight secondary ion mass spectroscopy (TOF-SIMS), X-ray diffraction (XRD), X-ray photoelectron spectroscopy (XPS), scanning and transmission electron microscopies (SEM and TEM), atomic force microscopy (AFM), energy dispersive X-ray spectroscopy (EDS/X), thermogravimetric analysis (TGA), differential scanning calorimetry (DSC), and electroanalytical methods which include linear sweep (LSV) and cyclic (CV) voltammetry and electrochemical impedance spectroscopy (EIS).

## 1. Introduction

Nanomaterials and nanotechnology have recently attracted enormous attention in recent research, thanks to their fascinating biological, chemical, and physical properties which led to their usage in different research fields such as material science, physics, biochemistry, and chemistry [1–5]. In definition, nanomaterials are materials with dimensions between 1 nm and 100 nm. In comparison to their bulk materials, size, purity, surface properties, stability, solubility, and shape are much improved, and these led to their usage in various applications [6]. In addition, nanomaterials composed of several layers which include the surface layer which may be functionalised easily, shell layer which differs from the core of the materials, and core layer which is the central part of the nanomaterial [7]. Furthermore, nanomaterials are categorized according to the material composition, and these categories include [4–7] (1) carbon-based which are predominantly carbon (carbon nanotubes (CNTs), fullerenes (C60), graphene, and carbon onions), (2) inorganic-based (metal oxides or sulphides), (3) organic-based which are made mostly from organic matters excluding the carbon-based, and (4) composite-based which are poly- or multiphase nanomaterials consisting of carbon-based coordinate to inorganic base or organic base. Interconnection of those pristine materials brings synergy to the nanocomposite. Among these categories, 2-dimensional (2D) nanomaterials which include graphene, hexagonal boron-nitride (h-BN), tungsten disulphide (WS_2_), and molybdenum disulphide (MoS_2_) and their nanocomposites (3D) have been mostly explored and applied at various applications because of their characteristics such as highly stable, large surface area, highly catalytic, and adsorption properties [8–12]. From the above mentioned 2D nanomaterials, MoS_2_ has recently gained much interest in research for its outstanding and exceptional chemical, optical, mechanical, electronic, and sensing properties [13–15]. MoS_2_ as one of the transition-metal dichalcogenides (TMDCs) consists of few monolayer of sheets, and each monolayer is held together by weak van der Waals forces [16, 17]. Depending on the type of synthetic route and conditions, MoS_2_ can be synthesised in different morphologies such as nanosheets, nanodots, nanorods, nanofibers, nanowires, and nanoonions [17–19]. These different morphologies lead to MoS_2_ nanostructures with different properties which are compatible for certain applications. In order to confirm successful preparation and determine the properties of MoS_2_ nanostructures and nanocomposites, sophisticated analytical tools are needed to characterize and analyze their surface and structural compositions. Most common characteristics that are studied to confirm the successful preparations are particle size, shape, and size distribution, concentration, agglomeration, surface characteristics, the existence of surface coatings, the occurrence of impurities, and structure [20–25]. Herein, we describe extensively the use of different methods for the characterization of MoS_2_ nanostructures. These methods include optical, morphological, structural, and thermodynamic instruments such as ultraviolet-visible spectroscopy (UV-vis), photoluminescence spectroscopy (PL), Fourier transform infrared spectroscopy (FTIR), Raman spectroscopy, time-of-flight secondary ion mass spectroscopy (TOF-SIMS), X-ray diffraction (XRD), X-ray photoelectron spectroscopy (XPS), scanning and transmission electron microscopies (SEM and TEM), atomic force microscopy (AFM), energy dispersive X-ray spectroscopy (EDS/X), thermogravimetric analysis (TGA), differential scanning calorimetry (DSC), and electroanalytical methods which include linear sweep (LSV) and cyclic (CV) voltammetries and electrochemical impedance spectroscopy (EIS). Representative results for MoS_2_ nanostructures and nanocomposites will be used to explain the information which can be deduced from different techniques.

## 2. Brief Fundamental of Molybdenum Disulphide

### 2.1. Structure of Molybdenum Disulphide

**Figure 1:**
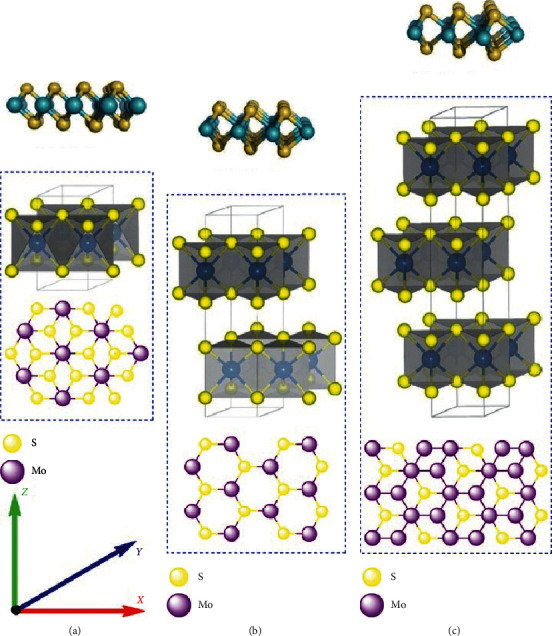
Illustration of the (a) octahedral IT, (b) trigonal prismatic 2H, and (c) trigonal prismatic 3R-crystal structures of MoS_2_ [36].

MoS_2_ is a type of transition-metal dichalcogenides (TMDCs); thus, it has a general formula, MX_2_, which is formed from the linkage between a d-block metal (*M* = W, Mo, Ti, and Zr) and two chalcogen atoms (*X* = S, Se, and Te) [14, 26, 27]. The Mo atom in MoS_2_ is held chemically coordinated between the two sulfur atom, S-Mo-S, forming a sandwiched-like structures [28–31]. As mentioned above, MoS_2_ is a 2D nanomaterial, and the layers on MoS_2_ are held together by weak van der Waals bonds forming a trigonal prismatic or octahedral structure [32, 33]. Generally, MoS_2_ forms three crystal structures which are one octahedral 1T structure and two trigonal prismatic structures, 2H and 3R (Figure 1) [34–36]. 2H phase is the naturally occurring stable form of MoS_2_ than metastable-stable nonnatural 1T and 3R phases [35]. However, upon annealing, electric doping, and by controlling strains, 2H-MoS_2_ can be transformed to 1T and 3R phases [36]. Due to the unique 2D structures, MoS_2_ nanomaterials possess extraordinary electronic, optical, mechanical, and thermal properties, and this made it gain popularity in applications such as lubricants, catalysis, sensing, photoluminescence, and optics [37–40].

### 2.2. Synthesis of MoS_2_

Various synthetic routes which include bottom-up and top-down methods have been used in the fabrication of MoS_2_ nanomaterials [36, 41]. Top-down approaches which include liquid-phase exfoliation and micromechanical exfoliation involve processes such as dispersion, sonication, and purification to exfoliate MoS_2_ from the bulk MoS_2_ [42]. Bottom-down approaches use Mo and sulfur precursors to synthetically produce MoS_2_ nanomaterials. The two most common bottom-up approaches used are hydrothermal and chemical vapor deposition (CVD) [43]. The hydrothermal method is the mostly used method because of its ease operability and large-scale synthesis. During the hydrothermal method, Mo precursors typically ammonum molybdate, (NH_4_)_6_Mo_7_O_24_·4H_2_O and sulfur precursor potassium thiocyanate, KSCN or thiourea, and CH_4_N_2_S are allowed to react in a Teflon autoclave at higher temperatures and pressure to yield MoS_2_ nanomaterials with controlled sizes and morphologies [44, 45]. CVD involves gaseous constituents with sulfur gas, H_2_S, or S_8_, allowed to interact with solid state Mo substrate, MoO_3_ [46, 47]. During the sulfurization process, sulfur flows to sulfurize on the Mo surface to make MoS_2_, and this yields a nanomaterial with uniform and thin films [36].

## 3. Structural, Morphological, and Thermal Characterization Techniques for Nanomaterials

### 3.1. Ultraviolet-Visible Spectroscopy

Ultraviolet-visible (UV/vis) spectroscopy is a form of spectroscopy which is based on the absorption, reflection, diffraction, scattering, and refraction properties of an analyte and uses electromagnetic radiation with wavelengths between 190 nm and 800 nm [48, 49]. The electromagnetic radiation can be divided into two regions, in which region between 190 nm and 400 nm and 400 nm and 800 nm corresponds to ultraviolet and visible, respectively [50]. During the analysis, there is absorption of light that causes the change in the electronic energy levels within the material; hence, sometimes it is called electronic transition spectroscopy [51]. The change in energy levels is due to the transfer of electron from the bonding orbital to the antibonding orbital [52]. In principle, the light from the source, usually xenon arch or mercury lamp interacts with the sample (contained in the cuvette). The sample absorbs light, and there is excitation of valence electrons. The detector then records the ratio of intensities between the blank and sample [53]. The ratio of intensities recorded by the detector is referred as transmittance *T* and is given by the following equation [54, 55]:

(1) $$\begin{array}{c} T=\frac{I}{I_{0}}, \end{array}$$

where *I* is the transmitted intensity, and *I*_0_ is the original/reference intensity. The transmittance can then be related to the absorbance *A* according to the following equation [55]:

(2) $$\begin{array}{c} A=-\log\;T. \end{array}$$

Most importantly, the UV/vis spectroscopy adheres to Beer–Lambert's law which says that the absorption of incident light is relative to the number of absorbing molecules present in the solution [55, 56]. Beer–Lambert's expression equation (3) can be written as

(3) $$\begin{array}{c} A=\varepsilon cl, \end{array}$$

where *ɛ* defines the molar extinction coefficient (dm^3^·mol^−1^·cm^−1^), *c* is the concentration (mol·dm^3^) of the absorbing species, and *l* represents the optical path length or dimension (cm) of the glass cuvette.

**Figure 2:**
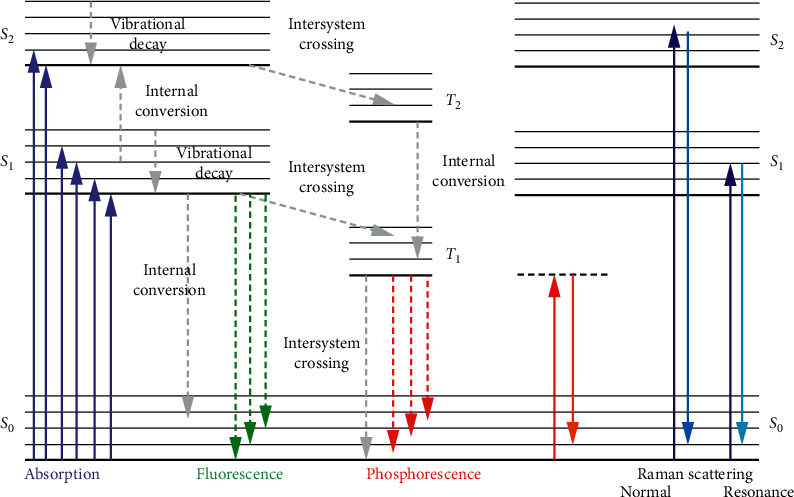
Jablonski diagram showing different types of electronic transitions (absorption, emission, and scattering) that can take place accompanied with different processes [58].

As explained above, when the incident light interacts with the sample, several transitions take place. The most favourable shift is from the lower energy orbital where unexcited electrons can be found and known as highest occupied molecular orbital (HOMO) to the highest energy orbital referred as lowest unoccupied molecular orbital (LUMO) [57]. Various transitions for organic-based nanomaterials are depicted by Jablonski diagram as shown in Figure 2 [58].

For inorganic-based nanomaterials, the transition that can occur is the d-d transition between the metal ions and ligand-to-metal-charge transition. As nanocomposites have both organic and inorganic constituents, their UV spectra can have transitions from both materials. Apart from the electronic transitions, UV/vis can be used to evaluate the band gaps of semiconductors or nanomaterials and nanocomposites with semiconductor behaviour. In addition, successful preparation of nanocomposite can be observed by looking at different types of shifts. These shifts include (1) auxochrome (substituents that do not absorb light but their presence shift the absorption maxima to longer wavelength), (2) red shift or bathochromic (shift towards longer wavelength), (3) blue shift or hypsochromic (towards shorter wavelength), (4) hypochromic effect (decreases absorption intensity), and (5) hyperchromic effect (increases the intensity) [57].

**Figure 3:**
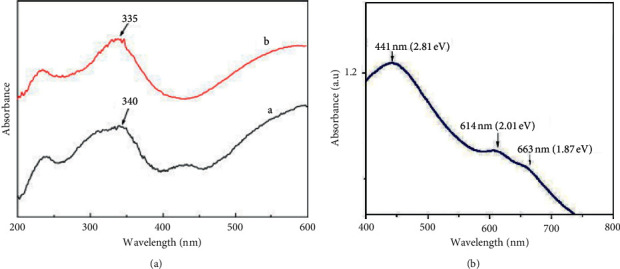
(a) Representative of UV/Vis of bulk MoS_2_ and MoS_2_ nanosheets [59] and the UV/vis (b) showing band gaps [60].

Li et al. [59] prepared MoS_2_ hollow spheres and obtained that UV/vis (Figure 3(a)) of hollow spheres (335 nm) blue shifted as compared to bulk MoS_2_ (340 nm). In another study, Chaudhary et al. [60] reported that MoS_2_ nanoflakes and UV/vis (Figure 3(b)) displayed two absorption peaks at 614 and 663 nm attributed to *K*-point excitonic transitions at the Brillouin zone. Their corresponding band gaps were 2.01 and 1.87 eV, respectively.

### 3.2. Photoluminescence Spectroscopy

**Figure 4:**
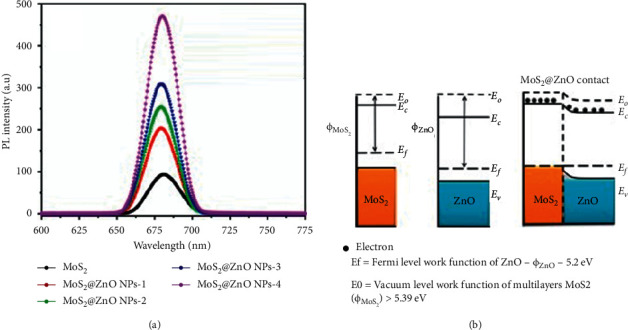
(a) PL spectra of MoS_2_ and MoS_2_@ZnO showing the enhancement of intensity when ZnO is incorporated in the MoS_2_ surface, and (b) schematic illustration of change in the charge generation and transfer process during composite formation [68].

Photoluminescence spectroscopy (PL) is a nondestructive and noncontact powerful technique which provides the information relating to the electronic structure of the nanomaterials [61–63]. From the photoluminescence spectra, electronic information such as band gap, recombination mechanisms, material imperfection, and impurities presence can be deduced [63–66]. The principle of PL spectroscopy can be explained using the Jablonski diagram (Figure 1). The laser beam is irradiated to the sample surface as in UV/vis spectroscopy) where upon interaction between the sample and light/beam, photoexcitation of electrons takes place [58, 66, 67]. During this photoexcitation process, electrons are excited from the ground state (*S*_0_) to the higher electronic states (*S*_1_, *S*_2_,…). At the higher electronic/excited states, the electrons can quickly relax to the lower excited (for example, from *S*_2_ to *S*_1_) or ground state (from *S*_1_ to *S*_0_) using processes such as vibrational decay/relaxation or internal conversion. Subsequently, another relaxation process, intersystem crossing in which electrons relax to triplet excited states (*T*_1_, *T*_2_,…), can take place. During these relaxation processes (vibrational decay, internal conversion, and intersystem crossing), the light is emitted in the form of fluorescence or phosphorescence [67]. The emitted photon is then measured, and the intensity is measured using the photodiode array detector.  Figure 4(a) shows the PL spectra of MoS_2_ and MoS_2_@ZnO nanocomposite with the excitation wavelength of 340 nm [68]. The PL of MoS_2_ shows the band peak at 681 nm attributed to the intrinsic emission or the band to band transitions in MoS_2_ nanosheets. The incorporation of ZnO NPs resulted in enhancement and blue shifting of the peak as a function of concentration of ZnO NPs. The enhancement and blue shift of the peak showed the successful deposition of ZnO NPs in the nanosheets of MoS_2_. The work function of the reported materials is shown in Figure 4(b), and MoS_2_ and ZnO exhibited the work functions of 5.39 eV and 5.2 eV, respectively. The difference in work functions signifies that electrons will be transferred from MoS_2_ nanosheets to ZnO NPs, while electron holes diffuse the other way.

### 3.3. Fourier Transform Infrared Spectroscopy

**Figure 5:**
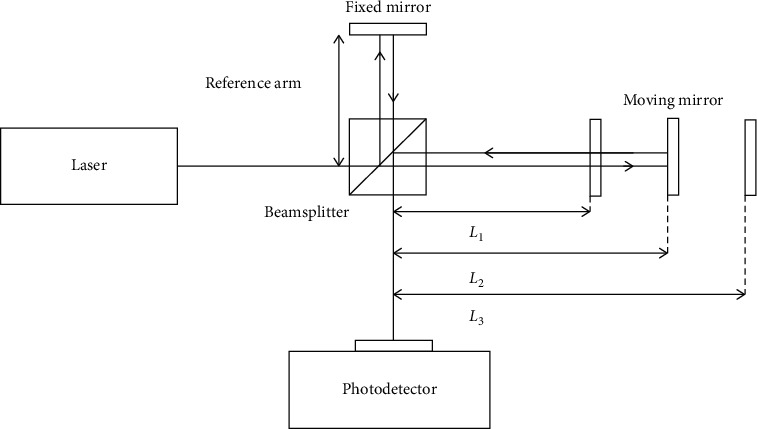
Illustration of the interferometer [75].

Fourier transform infrared (FTIR) spectroscopy is an analytical technique which provides the functional group, bonding type, and molecular conformation of organic and inorganic compounds [69, 70]. When the sample is irradiated with an infrared light source, the chemical bonds that held the atoms together absorb this radiation at frequencies that match their vibration modes unlike during UV/vis or PL spectroscopy where electrons get excited upon the absorption process. The vibration modes that can take place when light is irradiated include stretching, twisting, bending, rocking, wagging, or out-of-phase or in-phase deformation which occur between the wavenumbers of 400–4000 cm^−1^ [71]. The change in vibrational modes will then give rise to a spectrum with band, in which each spectral band represents different chemical environment [71]. The most important component on the instrumental setup of FTIR is the interferometer (Figure 5) which makes FTIR different from traditional IR dispersive spectroscopy [72]. The interferometer has fixed and movable/translating mirrors as well as the beam splitter. When light from a source comes, the interferometer splits it into two portions as illustrated in Figure 5. A portion of the splitted light is reflected to the fixed mirror and then redirected to the beam splitter where it goes to the detection system. The other light is reflected to the movable mirror and then back to the beam splitter and to the detector [73]. When these lights meet, constructive or destructive interference occurs [74].

**Figure 6:**
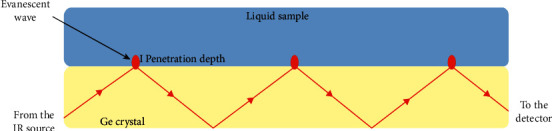
Setup for an ATR crystal [76].

**Figure 7:**
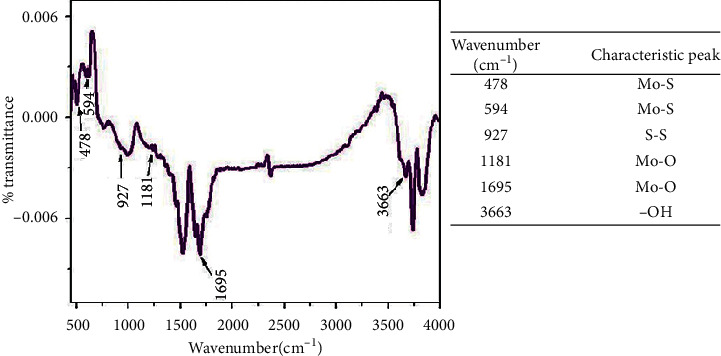
FTIR of pristine MoS_2_ nanosheet [60].

The FTIR have different operation modes such as the attenuated total reflection (ATR), nujol, and potassium bromide (KBr). Among these operational modes, ATR is mostly used as it avoids handling problems, sample preparation complexity, and spectral irreproducibility [6, 74]. This ATR-FTIR uses the total internal reflectance (TIR) of a crystal element such as Ge or diamond [76]. An illustration for an ATR element is shown in Figure 6. In principle, the sample is placed on an ATR crystal [74]. The IR beam from the light source enters the ATR element at a certain angle which corresponds to the critical angle between the sample and ATR element [76]. The light then undergoes TIR which forms an evanescent wave which extends beyond ATR [6, 74, 76]. The evanescent wave is absorbed by the sample, and it reaches the detector which generates the IR spectrum. The spectrum is a plot of % transmittance (%*T*) against the wavenumber *ṽ* (an inverse of wavelength, *λ*) [74]. According to the International Union of Pure and Applied Chemistry (IUPAC), it runs from high *ṽ* (small *λ*) to low *ṽ* (long *λ*) [74]. Thus, the wavenumber can be related to energy Δ*E* by the following equation:

(4) $$\begin{array}{c} \Delta E=hv=\frac{hc}{\lambda }=hc\bar{v}, \end{array}$$

where *v* is the frequency (Hertz), *c* is the speed of light (2.998 × 10^8^ m s^−1^), and *h* is the Planck's constant (6.626 × 10^−34^ J s). FTIR analysis of pristine MoS_2_ presented in Figure 7 shows several characteristic peaks attributed to different stretching/vibrations [60]. The vibration bands at 478 cm^−1^ and 594 cm^−1^ are attributed to Mo-S and the bands at 927 cm^−1^ are due to S-S bonds. The stretching vibrations at 1181 cm^−1^ and 1695 cm^−1^ are because of the hydroxyl group and Mo-O bonds, respectively. The peak at 3663 cm^−1^ also corresponds to the stretching vibration of the hydroxyl group.

The other type of FTIR which has recently found a great use is the FTIR microscopy (micro-FTIR). In this micro-FTIR, the IR spectrometer and optical microscopy are integrated together to allow the infrared beam to be focused on microscopic regions/area [77]. Micro-FTIR requires little sample preparation and can be used to identify microplastics on membrane filters [78]. Furthermore, it provides information in the micrometre range with the modification of the chemical composition [79]. The two common modes on micro-FTIR are transmission (gives high quality spectra but requires infrared transparent substrate; thus, there is difficulty in sample preparations) and reflectance (enable the rapid analysis of thick and opaque substrate) [77, 79]. Reflectance micro-FTIR is an alternative micro-FTIR technique having significant advantages over other FTIR techniques, such as easier sample preparation and the ability to share the same maceral area with standard reflected light microscopy [80]. The most common detector used in micro-FTIR is the focal plane array (FPA) detector, and it consists of elements whereby each element on the array represent the IR sensor allowing the measurement of wide field with relative ease [81–83]. FPA-micro-FTIR imaging technique reveals the distribution of chemical species with high resolution, and it is very fast as it records thousand spectra simultaneously within one single time-saving measurement [83].

### 3.4. Raman Spectroscopy

**Figure 8:**
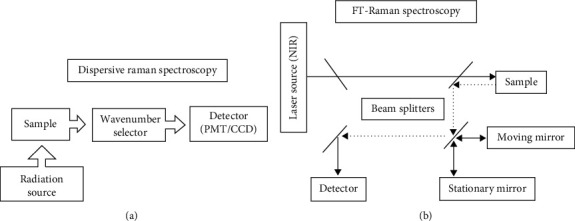
Basic instrumentation of (a) dispersive and (b) FT-Raman spectroscopy [88].

Raman spectroscopy is a form of vibrational, rotational, and low-frequency mode spectroscopy which gives information of the nanomaterial based on inelastic scattering measurements [84–86]. Raman spectroscopy was discovered in 1923 by Adolf Smekal, and scattered incidences were observed by CV Raman in 1928; hence, it is called Raman spectroscopy [87]. The two most common Raman spectrometer are dispersive and FT-Raman spectroscopy, and their instrumental setups are presented in Figure 8 [88].

**Figure 9:**
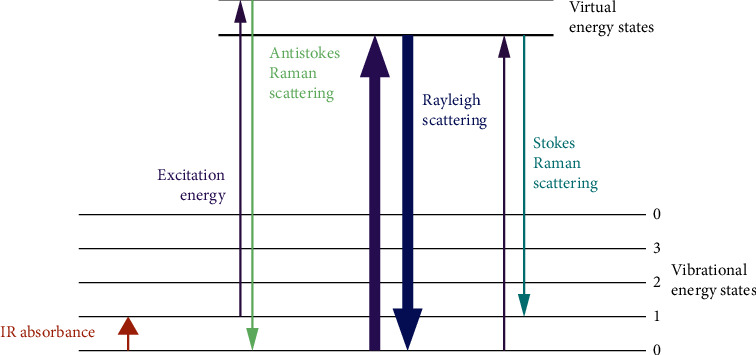
Diagram showing different types of scatterings [53].

Dispersive Raman spectroscopy uses a diffraction grating to disperse the scattered light from the sample. The light will then be detected upon multichannel detectors such as the photomultiplier tube (PMT) or charge-coupled device (CCD) [89]. Whereas in FT-Raman spectroscopy, the interferometer is used, and the light from a laser shines on the edge filter which directs it to different paths (lens) [90]. The light directed to lens 1 interacts with the sample and light from lens 2 goes to the slit of the spectrometer. The spectrometer consists of mirrors, collimating mirror which directs light to the grafting of the spectrometer. The grafting focuses the light to the focusing mirror and then to the charge-coupled device (CCD) which will generate the spectrum [90]. The spectrum generated results from the Raman effect occurred during interaction of the sample with light [86, 89]. The theory of the Raman effect is based on the inelastic scattering process which means that the photon's frequency in monochromatic light changes upon interaction with the substrate [53, 91]. During the interaction between light and sample, a virtual energy level is created Figure 9. Since the virtual state is a temporary state, the molecules will tend to relax to their ground state and that is when the scattering process occurs [92]. There are two types of scattering processes that can take place, elastic and inelastic scattering. During elastic or Rayleigh scattering, the molecule relaxes to its initial position releasing no photon. This kind of scattering is not considered as the Raman effect and is not observed on Raman spectrum. However, if the molecule relaxes to a different state or energy level, energy can either be lost or gained, and this type of scattering is known as inelastic scattering which can be divided into stoke or antistoke scattering. In stoke scattering, the molecule falls to a higher energy state as compared to its initial state, and during antistoke scattering, it falls to a lower energy state as shown in Figure 7.

**Figure 10:**
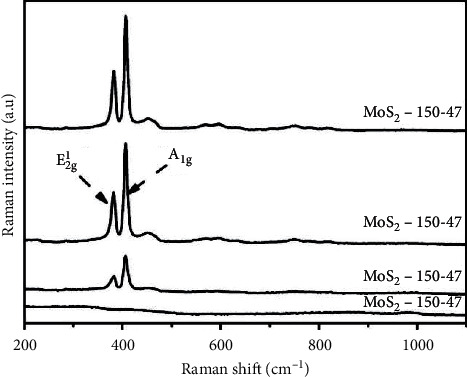
Typical Raman spectra of MoS_2_ [45].

Typical MoS_2_ Raman spectra Figure 10 shows two distinct Raman peaks at around 382 cm^−1^ and 406 cm^−1^ as reported by Luo et al. [45]. The peak at 382 cm^−1^ is attributed to Mo-S in-phase (E^1^_2g_) and at 406 cm^−1^ corresponds to out-of-phase (A_1g_) of Mo-S. From the results presented in Figure 10, it can be deduced that the intensity of the Raman spectra was enhanced by increasing the synthetic temperature and time.

There are other types of Raman spectroscopy (advanced) which are currently used, and these spectrometers include micro-Raman, resonance Raman spectroscopy (RRS), and surface-enhanced Raman spectroscopy (SERS) [89]. In micro-Raman spectroscopy, a special Raman microspectrometer which is a standard Raman spectrometer coupled with an optical microscope is used [93, 94]. This microspectrometer allows acquisition of Raman spectra and careful control of structural homogeneity of the microscopic sample which is difficult to acquire using standard Raman spectroscopy [94]. Micro-Raman has been widely used in biochemical and medicinal studies as it has a potential to detect changes in biochemical reactions and can be used as therapeutics and drug detection [95]. For RRS, the wavelength of the exciting laser matches with an electronic transition of the molecule [92]. This gives the spectra with enhanced/more intense peaks. In SERS, the sample is adsorbed on the surface of metal surface such as silver, gold, or copper [96]. This improves the Raman signals and also quenches the fluorescence caused by cutting agents, diluents, and matrices [89].

### 3.5. Time-of-Flight Secondary Ion Mass Spectroscopy

**Figure 11:**
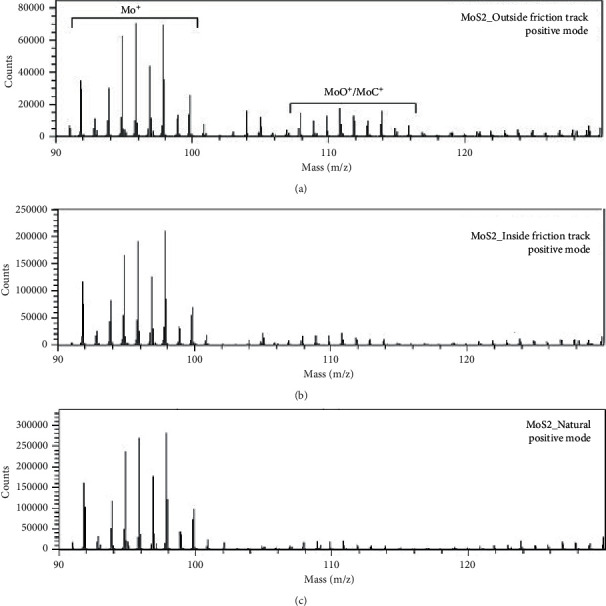
Natural, inside friction, and outside friction track TOF-SIMS spectra of MoS_2_ in the positive mode [102].

Time-of-flight secondary ion mass spectroscopy (TOF-SIMS) is a very sensitive surface technique that allows the determination of mass (mass analyser) of trace elements at very low concentrations [97, 98]. TOF-SIMS works on the sputtering process [99, 100]. In principle, the pulse of primary ions beams of high energy is bombarded onto the surface of an analyte (sample). The collision of primary ion beam with the sample surface produces the secondary ions. The resultant secondary ions are accelerated to the flight tube, and their mass are then measured when they reach the detector. In terms of operational modes, TOF-SIMS uses two operational modes, the static and dynamic modes [100]. In static mode, the detection limit is 10^9^ atom/cm^2^, and it obtains information in a few outer monolayers [101]. Whereas in the dynamic mode, high dose of ions is used, and this allows determination of elemental composition as a function of sputter depth [100, 101]. Colas and coworkers [102] investigated the chemical rearrangement of MoS_2_ under tribological conditions using TOF-SIMS, and the results obtained are presented in Figure 11. From the positive mode investigation, the natural MoS_2_ powder shows the Mo^+^ isotopic pattern with no evidence of contamination, whereas the analysis of MoS_2_ outside the friction track shows the appearance of MoO^+^ and MoC^+^. The MoO^+^ signifies the oxidation which is caused by the interaction between MoS_2_ and residual water during the sputtering process. Subsequently, the analysis inside the friction track showed the decrease in MoO^+^ and MoC^+^.

### 3.6. X-Ray Diffraction

X-ray diffraction (XRD) is the most significant characterization technique which is used to reveal the structural properties of the nanomaterials based on their diffraction pattern. XRD diffractometer consists of the X-ray cathode ray tube, filter, sample holder, and X-ray detector [103, 104]. In principle, the X-ray is produced by the cathode ray tube after its filament was heated at high voltage to produce electrons [103, 105]. The electrons accelerate and move towards the metal anode or target, bombarding with it [105]. This results in dislodging the electron from the core orbital of the materials. Relaxation of electrons to the anode releases X-ray [103]. The generated X-rays are filtered to produce monochromatic radiation which interacts with the sample [25]. The radiation is then passed to the detector, where it records signals to a count rate, which is known as the diffraction pattern [103]. The position of the patterns can tell about the nature and structural composition of a nanomaterial. The XRD diffraction pattern depends on the lattice parameter of the nanomaterials according to the following Bragg's law equation [103, 106, 107]:

(5) $$\begin{array}{c} 2d_{hkl}\sin\;\theta =\lambda , \end{array}$$

where *d*_*hkl*_ represents the interplaner spacing of the crystal, *θ* defines the diffraction angle, and *λ* is the wavelength usually of CuK*α* radiation = 1.5418 Å. *d*_*hkl*_ is given by

(6) $$\begin{array}{c} d_{hkl}=\frac{a}{\sqrt{h^{2}+k^{2}+l^{2}}}, \end{array}$$

where *a* is the unit cell size, and *h, k,* and *l* are the miller indices. XRD can also be used to determine the particle size of the nanomaterial [7]. This is performed by considering the following Debye Scherer formula:

(7) $$\begin{array}{c} D=\frac{0.89\lambda }{\beta \;\cos\;\theta }, \end{array}$$

where *D* defines the particle size (nm), and *β* is the full width at half maximum.

**Figure 12:**
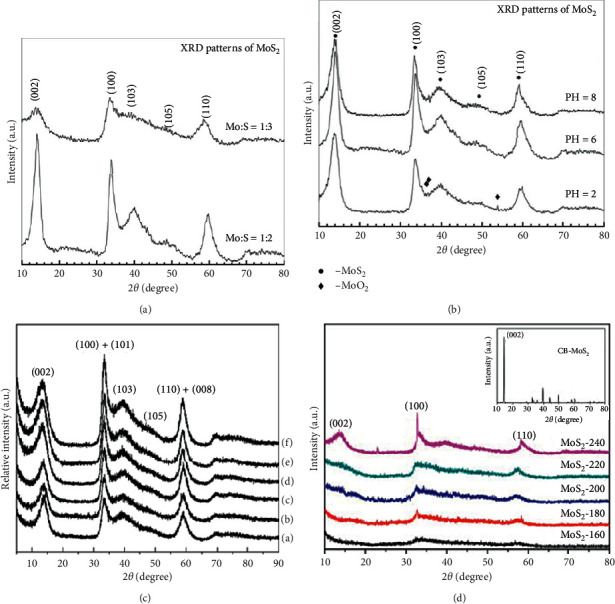
XRD pattern of MoS_2_ obtained from different (a) Mo/S mole ratio [109], (b) pH [109], (c) pressure [110], and (d) temperature [17].

The XRD pattern of a nanomaterial can be easily influenced by different synthetic conditions such as pH, temperature, and pressure. Cui et al. [109] demonstrated the effect of Mo/S mole ration and pH on MoS_2_ nanostructures. They found that the XRD pattern (Figure 12(a)) resembled the crystal structure of 2H-MoS_2_ when the mole ratio is 1 : 2. Decreasing the mole ratio to 1 : 3 resulted in broadening of XRD patterns. Increasing the pH caused the XRD peak (Figure 12(b)) to sharpen and enhance. In another study, Choi et al. [110] and Wang et al. [17] investigated the effect of pressure and temperature, respectively. From the X-ray diffraction (XRD) studies (Figures 12(c) and 12(d)), there was an enhancement of diffraction peaks with increase in pressure and temperature. From the studies, it can be deduced that pH, pressure, and temperature play an important role in determining the crystallinity and purity of MoS_2_.

### 3.7. X-Ray Photoelectron Spectroscopy

**Figure 13:**
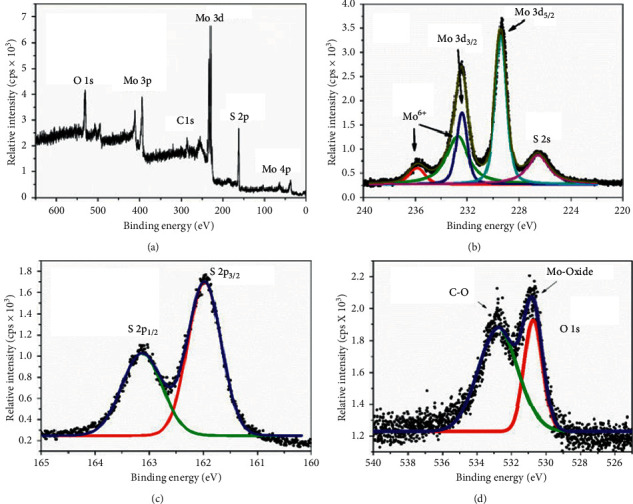
(a) XPS spectra of MoS_2_ showing the doublets Mo 3d peak and S 2p peak and high resolution spectra of (b) Mo 3d peak doublet and (c) S 2p peak doublets. (d) High resolution spectrum of O 1s peak [115].

X-ray photoelectron spectroscopy (XPS) also referred as electron spectroscopy for chemical analysis (ESCA) is a very suitable surface analysis technique developed in the 1960s by Siegbahn and coworkers for investigating the qualitative atomic composition and chemistry of a material [99, 111, 112]. Interestingly, XPS can also be used for detection of binding state/energies of substances, and variation in the binding state/energies can be used to identify the chemical state of the element [99]. In XPS principle, the flat specimen or sample is bombarded with X-rays with a well-distinct energy, and the X-rays interact with the inner electrons existing around the nucleus [113]. The electrons are then emitted at a well-defined kinetic energy, and *E*_K_ is illustrated by the following equation [113, 114]:

(8) $$\begin{array}{c} E_{K}=hv-E_{b}-\phi , \end{array}$$

where *hv* is the irradiated X-ray photon energy, *E*_*b*_ represents the electron binding energy, and *ɸ* defines the work function of the sample. From the equation, it can be deduced that the kinetic and binding energies of the emitted electrons depend on the irradiated photon energy on the surface of the sample [64]. A photoelectron spectrum which shows the elemental identity, chemical state, and quantity of the detected element is recorded by counting ejected electrons over a range of electron kinetic energies [64]. A typical XPS spectrum of MoS_2_ which proves that the elements of Mo and S are in the chemical state of MoS_2_ is shown in Figure 13 [115]. From Figure 13(a), it can be seen that MoS_2_ exhibits very strong Mo and S peaks attributed to the Mo 3d and S 2p with the binding energies of ∼230 and ∼1.62 eV, respectively. In addition, Mo 3p, S 2s, and Mo 4p peaks are observed. High resolution on Mo 3d (Figure 10(b)) reveals that Mo exhibits the oxidation state of 4+ due to doublet caused by *l-s* coupling. The high resolution on S 2p also shows a doublet which is due to the formation of sulphide (S 2p_3/2_ and S 2p_1/2_ peaks with the binding energies of 161.97 and 163.13 eV, respectively). The two doublets obtained show the successful synthesis of MoS_2_. Figure 10(d) shows the presence of Mo-O peak at ∼532.75 eV which shows the presence of MoO_3_ which is also formed during formation of MoS_2_.

### 3.8. Scanning Electron Microscopy

**Figure 14:**
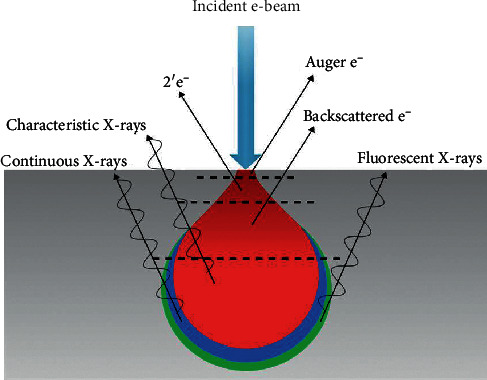
Illustration of sample-SEM signal interaction during analysis [119].

Scanning electron microscopy (SEM) is a form of microscopy consisting of column and the cabinet, widely used for surface morphology of specimen [116]. The analysis of surface morphology is achieved by allowing highly concentrated electron beam (produced in the column filament) to scan the surface of the sample. The detector receives the electric signal which is received by cabinet for quantification and quantitation. The cabinet analyses the information and gives results in the form of images. SEM produces images of high resolution in a size of less than 1–5 nm [25, 105]. This is due to the principle SEM use for analysis. During SEM analysis, the electron beam scans the specimen across its surface. The interaction between the electron bean and specimen results in emission of different signals which are collected and processed by the detector (Figure 11) [117]. SEM instruments detect three signals (Figure 14) which are secondary electrons (SEs) for characterization of specimen, backscattered electrons (BEs) for atomic number, and Auger electrons for the luminescence property of the specimen [118, 119]. The interaction of sample with SEM signals is shown in Figure 14. The two SEM techniques that are used to characterize the specimen are the field emission scanning electron microscopy (FESEM) and the environmental scanning electron microscopy (ESEM) [120]. The FESEM produces clearer, less electrostatically distorted images, with a spatial resolution/magnification better than the one of traditional SEM. The ESEM is widely used to analyze specimen that are “wet,” uncoated, or both by allowing for a gaseous environment in the specimen chamber [121].

**Figure 15:**
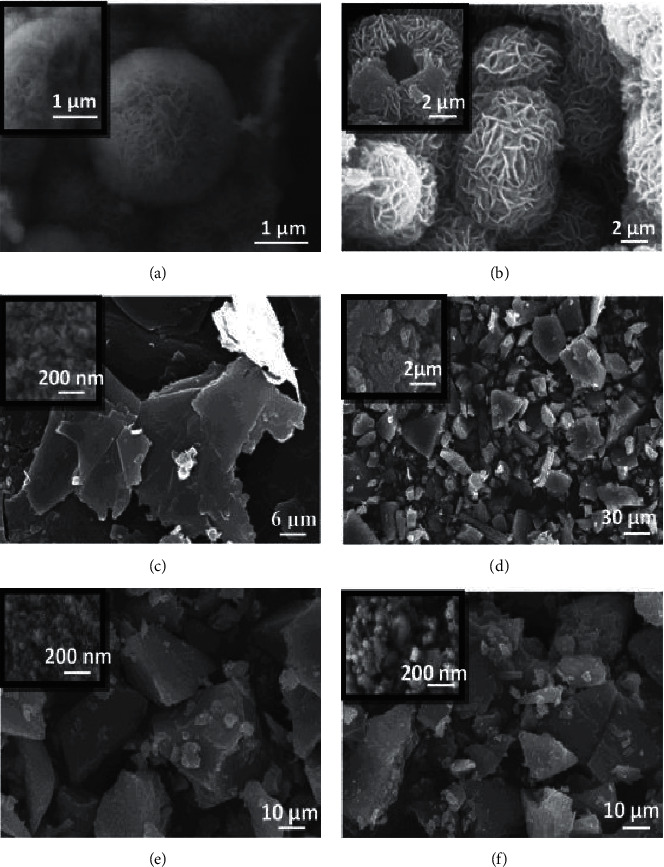
SEM micrographs of MoS_2_ obtained from surfactant-assisted hydrothermal methods [122].

Representative SEM micrographs of MoS_2_ nanostructures obtained from a simple hydrothermal method with the addition of surfactant (citric acid or ascorbic acid) reported by Zheng et al. [122] are shown in Figure 15. Micrographs obtained were flower-like hollow spheres (Figures 15(a) and 15(b)), micron sheet (Figures 15(c) and 15(d)), and blocky structures (Figures 15(e) and 15(f)). The flower-like MoS_2_ hollow sphere showed good crystallinity consisting MoS_2_ nanosheets with a thickness of about 10 nm.

### 3.9. Transmission Electron Microscopy

**Figure 16:**
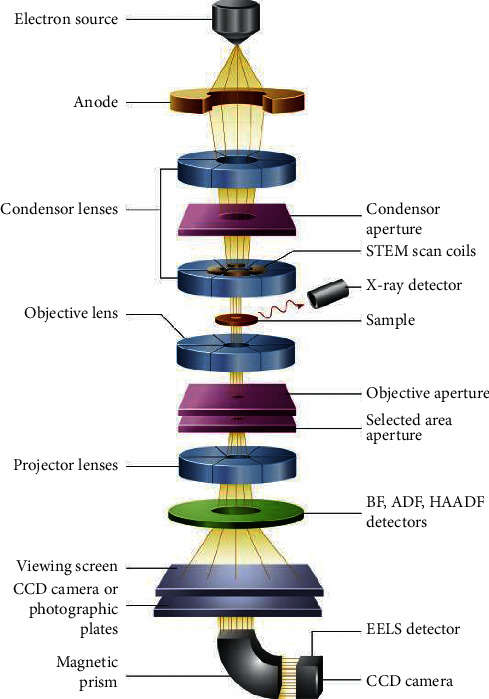
Basic components of TEM [125].

**Figure 17:**
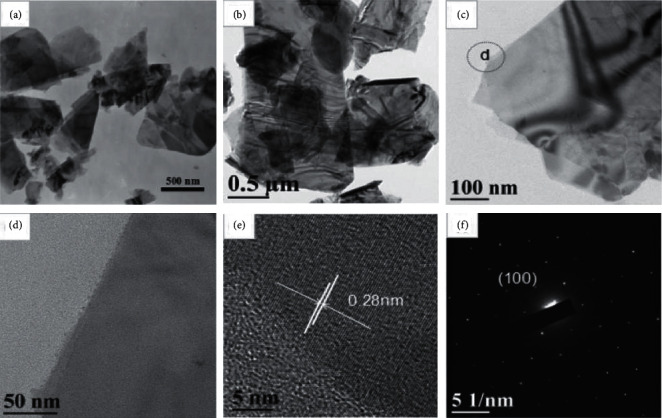
(a) and (b) TEM images of MoS_2_ nanosheets and (c) TEM image of nanosheets stacked together. (d) and (e) HRTEM image of the circle area marked in (c) and the corresponding SAED pattern (f) to (d) [127].

Transmission electron microscopy (TEM) depends on the penetration of electron beam into the crystalline sample rather than absorption as in SEM [105, 123, 124]. TEM consists of an electron emitting source, accelerator, condenser and objective lens, sample holder, and multiple-lens projector as shown in Figure 16 [125]. In principle, the electron beam from an electron gun which is sufficient to penetrate through the sample is incident onto the surface of the sample. Before reaching the sample, the electrons enter the condenser lens which focuses the electrons into controlled diameter and convergence [124–126]. The focused beam interacts with the sample, and X-ray emitted can be used for elemental composition (Section 3.11 for energy X-ray dispersive Spectroscopy (EDS or EDX)) [124]. Beam that penetrated through the sample goes to the objective lens which delivers it to the projector lens. From projector lens, it gets detected by the detector which gives imaging. This microscope system gives morphological and structural composition as well as crystallographic data of the nanomaterial after interaction and penetration of energetic electrons [7, 53]. Most importantly, TEM has several modes that can further reveal certain parameters. High-resolution TEM (HRTEM) reveals the crystallographic information of materials [105]. HRTEM utilizes the interference in the images of electron beam. In HRTEM, each electron interacts with the sample independently. In that case, after interaction, the electron passes to the imaging system where it undergoes phase changes. It then interferes with the image wave giving structural information. In addition to HRTEM, selected area electron diffraction (SAED) is another mode of TEM that can be used to give the lattice parameter of the nanomaterials [25]. SAED uses Bragg's law to give information on the interplanar spacing *d* as well as the crystal structure type. Examples of TEM and HRTEM of MoS_2_ are displayed in Figure 17 [127]. The TEM images of the exfoliated MoS_2_ nanosheets show very thin nanosheets in the nanometer scale (a), and the fringes resulted from the interference between the crystalline lattices of the individual sheets from stacking MoS_2_ nanosheets (b). The TEM images of MoS_2_ stacked together are shown in Figure 17(c), and their HRTEM (d) displays that the MoS_2_ nanosheets consist of at least two layers with smooth surface. HRTEM image of lattice fringes and SAED patterns shown as in Figures 17(e) and 17(f) indicate the single crystalline nature of the sheets. The lattice spacing of 0.28 nm (Figure 17(e)) is consistent with that of (100) planes.

### 3.10. Atomic Force Microscopy

**Figure 18:**
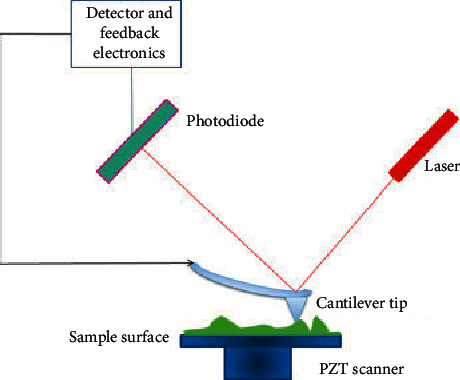
Illustration of the interaction between a sample and cantilever tip during AFM analysis [22].

**Figure 19:**
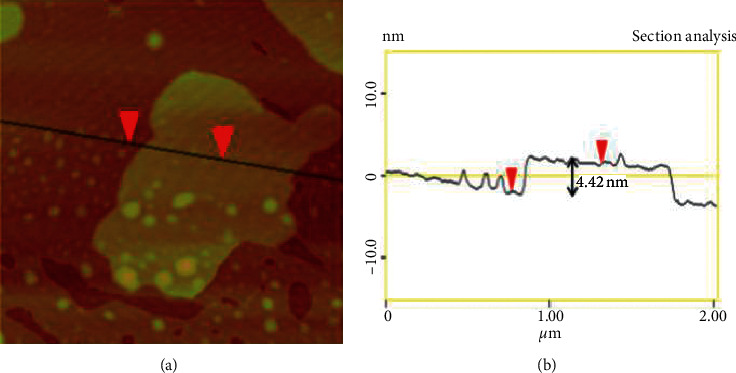
AFM image and height profile of MoS_2_ nanosheets [127].

Atomic force microscopy (AFM) is a versatile and powerful scanning probe microscopy that provides information such as physical topography, adhesion strength, magnetic forces, and mechanical properties about the surface of the sample [22, 128–130]. As compared to SEM and TEM, AFM gives reliable measurements at nanoscales [131]. Furthermore, AFM offers three-dimensional (3D) topographical images with higher resolutions unlike two-dimensional (2D) projections given by SEM and TEM [129, 130]. The operation principle of AFM is based on the interaction between the surface of the sample and cantilever tip (Figure 18) [22]. When the cantilever tip approaches the sample surface, a small deflection which is due to the attraction force between the surface and tip is observed [64]. The deflection on the tip creates a 3D image of the sample's surface topography and is monitored using a photodiode detector. The sample-tip interaction can occur using different modes which are contact, noncontact, and tapping. In the contact mode, the tip is in close contact with the surface of the sample, and then, information is acquired by monitoring the interaction forces, whereas in the noncontact mode, the tip hovers above the sample's surface and the attraction force between the sample and tip is measured. In the tapping mode, the noncontact and contact modes are combined, and the images obtained are of higher resolutions as compared to the latter and former [128–130]. This is achieved by oscillating the tip at its resonating frequency, and the tip is then allowed to impact (hit) the sample surface occasionally. During the impacting process, the information is then gathered.  Figure 19 shows the AFM image (a) and height profile (b) of MoS_2_ [127]. From the image, it can be deduced that the surface of the MoS_2_ nanosheets is very smooth and clean with uniform thickness across the lateral dimension. The height profile reveals that the thickness distribution is approximately 2 × 2 µm^2^ with the thickness of 4.42 nm.

### 3.11. Energy Dispersive X-Ray Spectroscopy

**Figure 20:**
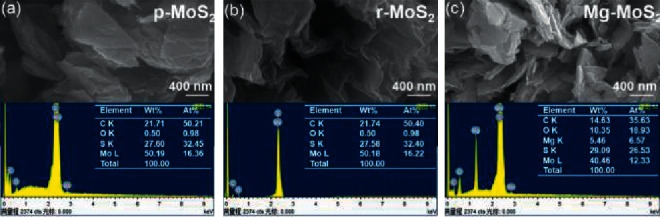
SEM and EDS of (a) pure MoS_2_, (b) restacked MoS_2_, and (c) Mg deposited MoS_2_ [134].

Energy dispersive X-ray spectroscopy (EDX or EDS) is an elemental analysis analytical technique coupled in SEM and TEM [7]. EDX or EDS can detect elements with atomic weight higher than that of boron [132]. In principle, a primary electron beam with energy between 10 keV and 20 keV interacts with the sample as in TEM and SEM. This causes excitation of electrons from the inner shell (nucleus) resulting in creation of electron holes or vacancies [25, 105]. The electrons in the higher energy states (outer shell) are then transferred to the electron vacancies releasing superfluous X-ray photons. By probing the energy released, the elements in the sample can be determined as different elements have distinct X-ray property [133].  Figure 20 shows the SEM and EDS analysis of pristine MoS_2_ (a), restacked MoS_2_ (b), and Mg intercalated MoS_2_ (c) [134]. The SEM images of all samples show that the MoS_2_ have a nanosheet-like shape. The elemental distribution of pure (a) and restacked (b) MoS_2_ shows the equal weight percentage of element presents. Upon intercalation of Mg (c), the weight percentages of Mo decreased and S increased with the appearance of Mg.

### 3.12. Thermogravimetric Analysis

**Figure 21:**
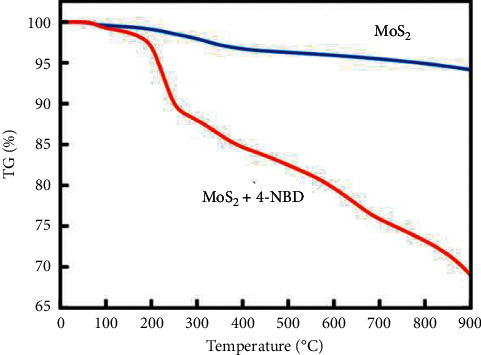
TGA thermogram of pure MoS_2_ and diazonium functionalised MoS_2_ [137].

Thermogravimetric analysis (TGA) is a form of thermodynamic analytical technique used to measure the change in the weight of a material with respect to temperature and time [25, 53]. The temperature is programmed, and the experiment takes place in a gas-controlled environment [53]. The change in mass/weight of nanomaterial may be due to several reasons which include evaporation of moisture or adsorbed solvents, oxidative decomposition, oxidation process occurring on metals, and thermal decomposition [135, 136]. Modern TGA instruments consist of a sample pan that is placed on a precise balance. The sample pan and balance are incorporated in a furnace. The sample pan is then heated in a furnace to a specific temperature in which the gas is flowing and exiting through an exhaust [53, 135]. The change in weight is recorded and displayed as a TGA curve. The TGA curve displays the results as a weight percent plotted against temperature and can give information such as thermal degradation reaction, enthalpy, heat expansion coefficient, phase transitions, and diagrams.  Figure 21 shows that the TG thermogram of pristine MoS_2_ and 4-nitrobenzenediazonium tetrafluoroborate (4-NBD) functionalized MoS_2_ [137]. The pristine and 4-NBD-MoS_2_ show a minimal mass loss at around 100°C, which is because of the loss of residual water and other small organic molecules adsorbed on the surface of the materials. For 4-NBD-MoS_2_, there is small mass loss below 200°C attributed to dissociation of van der Waals bonded molecules. The significant mass loss between 200°C and 300°C shows the total covalent bond breaking between MoS_2_ and 4-NBD and total degradation continuing at around 700°C.

### 3.13. Differential Scanning Calorimetry

Differential scanning calorimetry (DSC) is a technique that is used to study the melting/crystallisation behaviour, solid-solid reaction, and specific heat of a sample [53]. It can also be used to deduce the structure and stability of nanomaterials, as well as their conformation, as material transitions will vary as a subject of nanomaterial composition [138]. The technique measures the difference in heat flow between a reference and sample [139]. This differential heat flow is then recorded as a peak. The change in enthalpy is directly related to the area underneath the peak, and this is an indicative of thermal event (exothermic or endothermic) taking place [140]. The technique uses the principle of TGA; however, it measures heat flow as a function of temperature unlike the change in weight. Again, it differs from differential thermal analysis (DTA) which measures difference in temperature. Heat flow of the sample can be given by the following expression equation [141]:

(9) $$\begin{array}{c} \phi_{s}=\frac{T_{s}-T_{c}}{R_{\text{th}}}, \end{array}$$

where Φ_*1*_ defines the heat flow of sample, *R*_*th*_ is the thermal resistance, and *T*_*s*_ and *T*_*c*_ are the temperature of the sample and empty sample holder. The heat flow of reference is defined as

(10) $$\begin{array}{c} \phi_{r}=\frac{T_{r}-T_{c}}{R_{\text{th}}}, \end{array}$$

where *T*_*r*_ is the reference temperature. The signal given from the DSC is the difference between the heat flow of the sample and reference and can be written mathematically as

(11) $$\begin{array}{c} \phi =\phi_{s}-\phi_{r}=\frac{T_{s}-T_{c}}{R_{\text{th}}}-\frac{T_{r}-T_{c}}{R_{\text{th}}}. \end{array}$$

Rearranging the above equation simplifies the expression to

(12) $$\begin{array}{c} \phi =\frac{T_{s}-T_{r}}{R_{th}}. \end{array}$$

**Figure 22:**
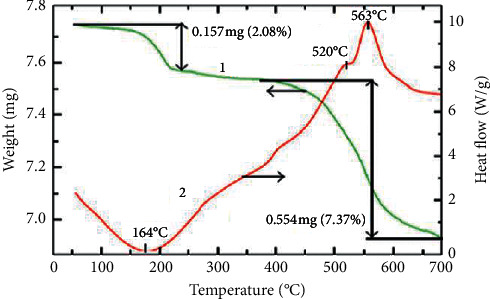
DSC-TGA plot of MoS_2_ [142].

Typical DSC-TGA curve of MoS_2_ is shown in Figure 22. The heating of molybdenum disulphide shows water loss and other adsorbed molecules below 200°C, and this is accompanied by the small endothermic effect (red) of sulfur trace melting. This effect can be due to prime disulphide decomposition into molybdenum and sulfur with subsequent oxidation of the latter and sulfur oxides loss. The weight loss of about 7.37% at higher temperatures shows the strong thermochemical behaviour and stability of MoS_2_.

## 4. Electrochemical Characterization of Nanomaterials

Nanomaterials exhibit chemical and physical properties which differ from those of their conventional bulk materials. One of the greatest differences is their large surface area which results in nanomaterials having enhanced reactivity as it was reported that decreasing the particle size (increasing surface area) results in enhancement in processes such as ion insertion/removal and electron transport process [143]. Spectrophotometric methods find it difficult to reveal those processes occurring on the surface of nanomaterials. In that instance, sophisticated methods are required to reveal those processes. Electrochemical/electroanalytical methods are a set of methods that can be used to study the surface reaction of nanomaterials when they interact with the conductive electrode [144]. This is performed by measuring parameters such as time, *t*; current*, i*; charge, *Q*; and potential, *E*. In this section, we will first look at the basic principle of electrochemistry to understand processes that are taking place for an electrochemical reaction to occur. Mass transport which helps in delivering the analyte to the electrode surface for the redox reaction to occur will be discussed briefly. This will be followed by exploring the three most used electroanalytical techniques, linear sweep voltammetry (LSV), cyclic voltammetry (CV), and electrochemical impedance spectroscopy (EIS).

### 4.1. Principle of Electrochemistry

#### 4.1.1. Overview of Electrochemical Cell

In definition, electrochemistry can be defined as a study that deals with interconvertion between electrical and chemical reactions, and the flow of electrons is related to the chemical change [145]. In this essence, the electron transfer process plays a central role in chemical reactivity of a substance. This electron transfer process takes place in an electrochemical cell consisting of pair of electrodes immersed in an electrolyte. The two electrodes are connected together by a conducting wire which permits the flow of electrons from one electrode to another. The flow of electrons results in a redox (reduction-oxidation reaction) occurring. The oxidation process (loss of electrons) occurring on the anode or metal *M* can be expressed as [146]

(13) $$\begin{array}{c} M⟶M^{n+}+ne^{-}. \end{array}$$

On the other side of the electrochemical cell (cathode), electrons are gained (reduction process) and given by the following equation [146]:

(14) $$\begin{array}{c} M^{n+}+ne^{-}⟶M. \end{array}$$

#### 4.1.2. Interfacial Processes on the Electrode Surface

In an electrochemical cell, we are more concerned about the processes that take place at the interface between the electrolyte (bulk solution) and electrode, normally referred as an electrolyte/electrode interface. In a modern electrochemical cell, there are three types of electrodes that assist in redox reaction occurring. Those three electrodes are [145]

  Working electrode (WE): an electrode where reaction takes place and the measurements are done on this electrode. Normally, researchers do electrodeposition of analyte on it. Normal WE are made of glassy carbon, mercury, and gold. Recently, there is a development of screen-printed electrode (SPE) made of the above materials. From experiment to experiment, the WE can be changed to provide different potential window.
  Reference electrode (RE): it has a well-defined and stable potential and used as a reference point of potential to the WE. The most commonly used REs are silver/silver chloride (Ag/AgCl), saturated calomel electrode (SCE), and standard hydrogen electrode (SHE).
  Counter electrode (CE): completes the cell by allowing the flow of the electrode. Its surface area is greater than the one WE, so that it cannot inhibit kinetics on the WE. Platinum wire and disk are the most typically used CEs.

At the electrolyte/electrode interface, the electrode that is involved is the WE. For an electrochemical reaction to proceed, there are three main processes that must take place:

1. Mass transport: delivers analyte to the electrode surface and then takes the product to the bulk solution
2. Faradaic: electrons are transferred across the electrolyte/electrode interface, and it is governed by Faraday's law which states that the amount of the chemical reaction caused by the flow of current is proportional to the amount of electricity passed
3. Non-Faradaic: processes such as adsorption (reactant) and desorption (product) that occurs on the electrode surface.

Considering the general reaction equation (15), where *O* and *R* represent oxidant and reductant, respectively, and ne-defining number of electron transfer,

(15) $$\begin{array}{c} O+ne^{-}⟶R. \end{array}$$

The interfacial electrode processes occur in a series of events which are as follows:

1. *O* must be successfully transported from the bulk solution to the electrode surface (mass transport)
2. *O* must then adsorb transiently onto the surface of electrode (non-Faradaic)
3. Charge transfer between the electrode and adsorbed *O* and *R* is formed (Faradaic)
4. *R* desorbs from the electrode surface (non-Faradaic)
5. *R* must then be transported from the electrode surface back into the bulk solution (mass transport).

**Figure 23:**
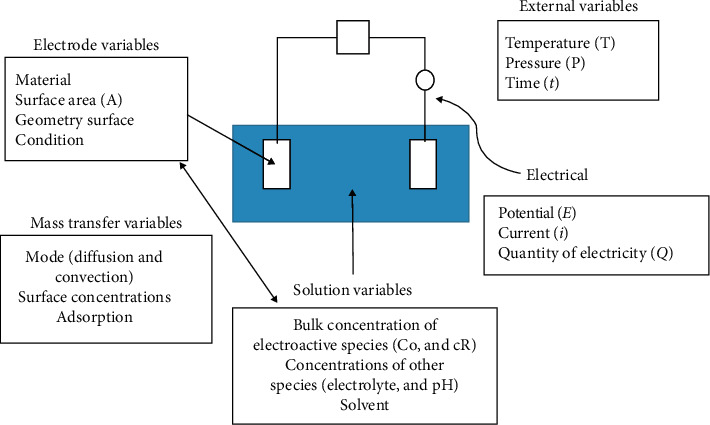
Factors that affect the interfacial process of the electrochemical reaction [147].

Several factors can affect these interfacial events, and summary of those factors is illustrated in Figure 23.

#### 4.1.3. Mass Transport and Electrochemical Reactions

Effectiveness of an electrochemical process requires constant supply of an analyte from the bulk solution to the electrode surface as well as the transport of product from the electrode surface to the bulk solution to restrict the recombination process since electrochemical reactions are reversible in nature. There are three modes of transport that can help in delivering the analyte to the electrode surface and carrying products to the bulk solution. These modes of transports include [146]

1. Diffusion: movement of analyte due to concentration gradient
2. Convection: movement due to mechanical force and
3. Migration: movement of charged species due to potential gradient

Most of the electrochemical reactions take place in a stagnant or quiescent solution which avoids movement via convection. Furthermore, the nature of the electrolyte (inert), which is employed, restricts the migration mechanism. Consequently, the most common mode of transport that occurs is the diffusion [146]. The diffusion of species is governed by Fick's laws (first and second) of diffusion which says the molar flux due to diffusion is proportional to the concentration gradient and is given by the following relation equations [148, 149]:

(16) $$\begin{array}{c} J_{i}=-D_{i}\left(\frac{\partial c_{i}}{\partial x}\right), \end{array}$$

(17) $$\begin{array}{c} \left(\frac{\partial c_{i}}{\partial t}\right)=D_{i}\left(\frac{\partial^{2}c_{i}}{\partial t^{2}}\right), \end{array}$$

where *J*_*i*_ is the molar flux, *D*_*i*_ is the diffusion coefficient, *C* is the concentration, and *x* is the distance to the electrode surface.

### 4.2. Voltammetry

**Figure 24:**
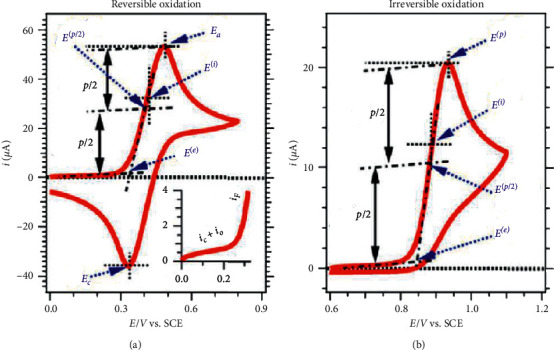
Voltammograms showing reversible (a) and irreversible (b) redox processes [150].

Voltammetry is one of the most significant electrochemical methods used to measure current-potential relationship on an electrode [144]. The applied potential is the driving force for an electrochemical reaction to take place in this technique and depends mostly on the electroactive species across the electrolyte/electrode interface. The current-voltage relation arises from the three-electrode system: WE, RE, and CE. In principle, the electroactive species in the electrolyte are drawn towards the WE via any form of mode of transport (diffusion, convection, and migration). The electroactive species adsorb on the surface of WE, and the half-cell redox reaction takes place. Another corresponding half-cell redox reaction occurs on the CE to complete the electron flow. The resultant current flowing is given in the plot which is the total reflection of electroactive species involved. The obtained plot of current-potential is called voltammogram in which the current is on the *y*-axis and applied potential is on the *x*-axis. Typical reversible voltammograms are displayed in Figure 24.

There are various forms of voltammetry techniques including linear sweep voltammetry (LSV), cyclic voltammetry (CV), potential step, stripping voltammetry, and differential pulse voltammetry. Among the abovementioned techniques, LSV and CV have found a widespread usage in electrochemical application such as hydrogen evolution reaction (HER) where they are used to monitor the HER parameters such as onset/overpotential, current density, reaction mechanisms, stability, durability, and the electrochemical active surface area (ECSA).

#### 4.2.1. Linear Sweep Voltammetry

**Figure 25:**
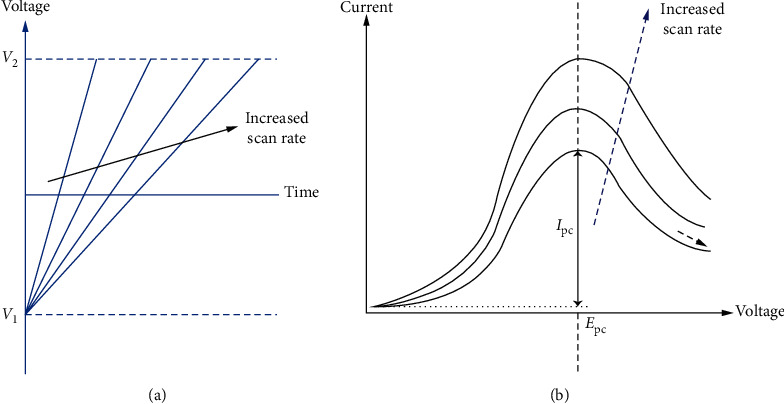
(a) Linear sweep wave form and (b) voltammogram at different scan rates [146].

Linear sweep voltammetry (LSV) is a type of voltammetry which involves scanning the potential of the WE linearly with time with a scan rate between 1 mV/s and 1000 mV/s [146]. The reduction and oxidation processes of an electroactive species are shown as a current signal at the potential where it gets oxidized or reduced. LSV is characterized by linear waveform (Figure 25) and the potential on the WE change linearly with time. The instantaneous applied potential at a given time *t* is described by the following equation [151].

(18) $$\begin{array}{c} E_{t}=E_{i}\pm vt, \end{array}$$

where *E*_*t*_ is the applied potential at a given time, *E*_*i*_ is the potential where redox reaction is not occurring, and *v* is the scan rate. The sign is influenced by the direction of the scan where negative is for cathodic sweep and positive is for anodic sweep. Representative linear sweep voltammogram is displayed in Figure 19.

**Figure 26:**
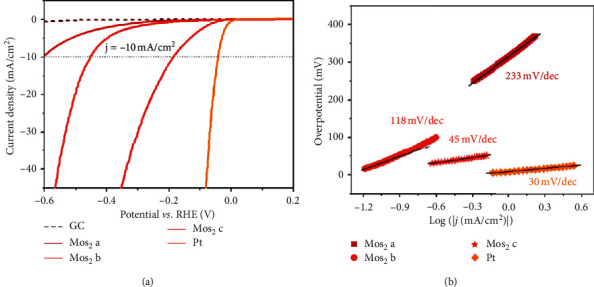
(a) LSV polarisation curves of MoS_2_ and commercial Pt electrocatalysts as well as their corresponding (b) Tafel plots [152].

LSV voltammogram can give information such as onset and overpotential of an electrocatalyst for HER. Most importantly, it can also be used for Tafel analysis. Wan et al. [152] engineered fractal-shaped MoS_2_ and used LSV (Figure 26(a)) to study their electrocatalytic activity using the GC working electrode. Commercial Pt showed excellent HER property needing only overpotential (*η*) of approximate 90 mV to drive a current density of 10 mA cm^2^. Its corresponding Tafel slope (Figure 26(b)) was 30 mV/dec which suggest that the Heyrovsky step was the rate-determining step. Fractal-shaped MoS_2_ electrocatalyst showed higher overpotentials and Tafel slopes.

#### 4.2.2. Cyclic Voltammetry

Cyclic voltammetry (CV) has become the most important and widely employed potentiodynamic electrochemical technique in many fields of electroanalytical chemistry [145]. It can be used to acquire information about the redox behaviour of nanomaterials [153]. CV is an extension of LSV where the current is measured between two potentials; thus, the potential scan is reversed [144, 149]. The applied potential is in a linear fashion as in LSV until switching potential *E*_*λ*_ at a switch potential scan *t*_*λ*_ is reached to start a backward scan. The overall CV process is given by the following expression equation:

(19) $$\begin{array}{c} E_{t}=E_{i}\pm vt_{\lambda }\mp v\left(t-t_{\lambda }\right)=E_{i}\pm 2vt_{\lambda }\mp vt. \end{array}$$

**Figure 27:**
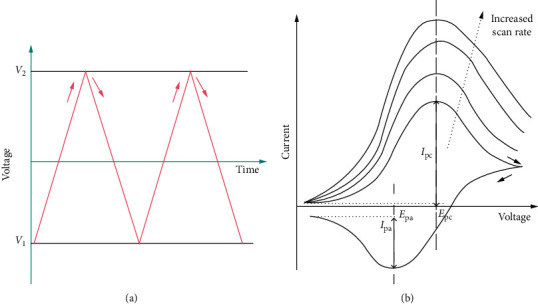
Representative (a) triangular wave form and (b) cyclic voltammogram as a function of scan rate [146].

It uses the triangular waveform (Figure 27(a)) and the potential scans from the initial set potential to the final set potential. Thereafter, the potential sweep is reversed to the opposite direction and scanned to return to the initial set potential to complete the cycle. The reversal scan can be applied several times generating multiple cycling voltammogram (Figure 27(b)).

**Figure 28:**
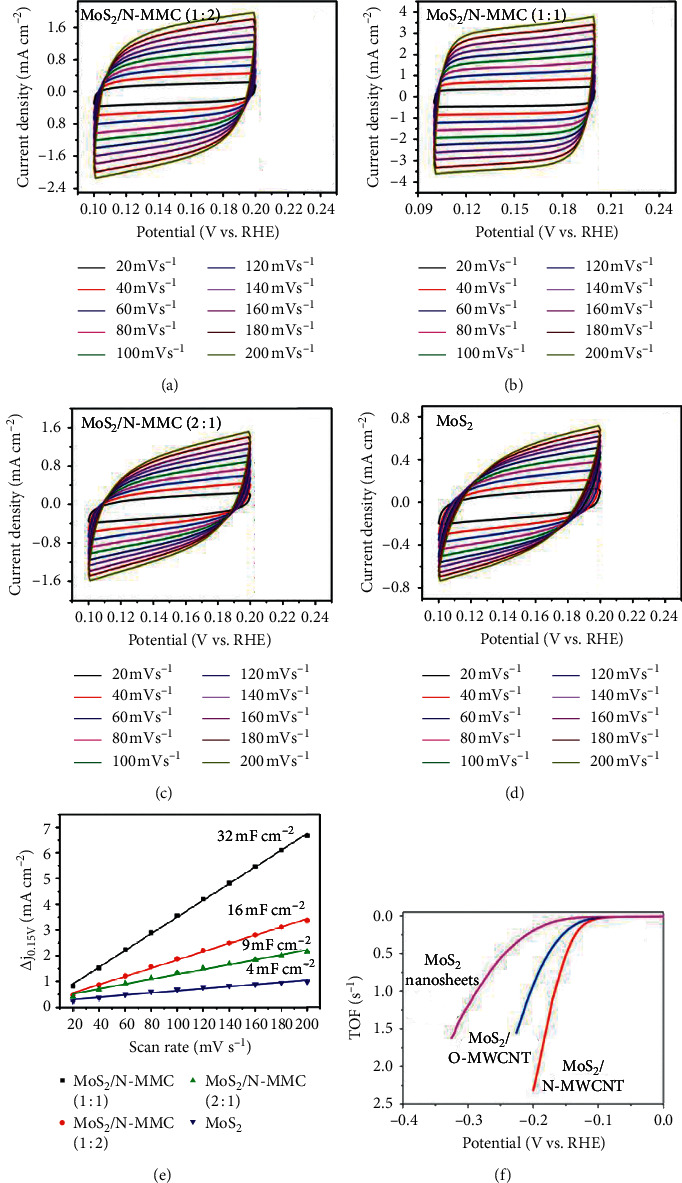
(a–d) Cyclic voltammogram of MoS_2_ supported N-doped macromesoporous and their corresponding ECSA results (e) [154]. (f) TOF plot of MoS_2_-multiwalled carbon nanotubes [155].

CV is useful in electrocatalysis by revealing the number of active sites presence. This is performed by determining the electrochemical active surface area (ECSA) as well as turnover frequency (TOF) of the material. ECSA is directly proportional to the capacitance double layer. It can be obtained from cyclic voltammogram by measuring the current response at different scan rates. Chen et al. [154] prepared MoS_2_ with N-doped macromesoporous carbon as an electrocatalyst for HER and determined the ECSA. They achieved it by measuring the current response at a potential range of 0.1 V-0.2 V at scan rates between 20 mV/s and 200 mV/s. Cyclic voltammograms of the as-synthesised electrocatalysts are shown in Figures 28(a)–28(d). The ESCA is obtained by plotting *∆j* (△*j* = *j*_a_ − *j*_c_) and 0.15 V as a function of scan rates and is displayed in Figure 28(e). The slopes obtained from a linear plot correspond to the capacitance double layer. In another study, Dai and coworkers [155] used CV to deduce the TOF values of MoS_2_ with multiwalled carbon nanotubes composites and results are shown in Figure 28(f).

### 4.3. Electrochemical Impedance Spectroscopy

**Figure 29:**
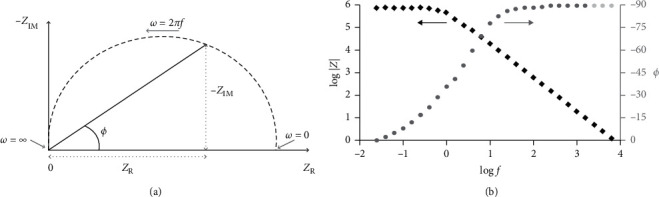
Representation of EIS (a) Nyquist and (b) Bode plots [162].

Electrochemical impedance spectroscopy (EIS) is the most effective and reliable methods to study the chemical and physical processes that takes place in the electrolyte/electrode interface [156, 157]. This is achieved by getting information on electrochemical characteristics such as double-layer capacitance, diffusion impedance, rate of charge transfer and charge transport processes, and solution resistance [158, 159]. EIS based on the application of an applied current (AC) to the system gives response as a function of the frequency [160]. EIS data are represented mostly using two different plots, Nyquist plot (Figure 29(a)) reporting *Z*″ as a function of *Z*′ and Bode plots (Figure 29(b)), which is a plot of a pair of graphs recording log |*Z*| and *θ* against log *f* [161, 162].

**Figure 30:**
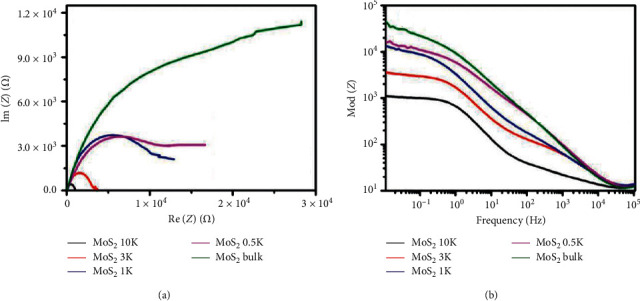
EIS (a) Nyquist and (b) Bode plots of MoS_2_ synthesised at different conditions [164].

From the EIS analysis, the charge transfer resistance *R*_ct_ can be determined and related to the interfacial electrochemical processes. The *R*_ct_ value obtained corresponds with how fast the HER process is, and ideally, it must be as low as possible as it shows that the electron transport ability is enhanced, and this leads to excellent HER activity [163]. For example, Benson et al. [164] used EIS analysis to evaluate the HER kinetics of MoS_2_ nanorods, and the obtained Nyquist and Bode plots are shown in Figure 30.

## 5. Conclusions

This review designated to look at the role of various techniques for the characterization of nanomaterials. Several conventional analytical methods can be used for the characterization of some features of nanomaterials, since nanomaterial properties usually differ from those of their bulk counterparts. Hence, in this comprehensive summary, we demonstrated the application of different analytical techniques (spectroscopic, microscopic, physical, and electrochemical), emphasizing on their application principle. Therefore, we finally hope that a careful reading of this review will help to identify which valuable techniques merit eﬀorts for further technical improvements. Spectroscopic characterization methods which probe the properties of nanomaterials by absorbing the light of a certain wavelengths can be used to reveal the optical studies which are useful for semiconductor and electrocatalytic applications as well as showing the different functional groups obtained during synthesis of nanomaterials. The other important analytical methods, which can reveal the phase properties of nanomaterials, such as the XRD are useful to calculate the crystalline sizes of the nanomaterials from the Debye–Scherrer equations. Microscopic techniques, which scan on the surface or transmit through the nanomaterials, are valuable for revealing the external and internal morphologies of the nanomaterials. Coupled with EDX detectors, microscopic techniques can show elemental composition on the nanomaterials. TGA and DSC (thermal methods) can be beneficial to probe the thermal properties of materials to understand the phase transitions. Finally, electrochemical methods have gained an interest in revealing the redox properties of the materials.
